# In Situ Formation of Copper Phosphate on Hydroxyapatite for Wastewater Treatment

**DOI:** 10.3390/nano12152650

**Published:** 2022-08-02

**Authors:** Fatemeh Rahmani, Arezoo Ghadi, Esmail Doustkhah, Samad Khaksar

**Affiliations:** 1 Department of Chemical Engineering, Ayatollah Amoli Branch, Islamic Azad University, Amol 4635143358, Iran; fatemehrahmani766@gmail.com; 2 Koç University Tüpraş Energy Center (KUTEM), Department of Chemistry, Koç University, Istanbul 34450, Turkey; 3 School of Science and Technology, The University of Georgia, Tbilisi 0171, Georgia; s.khaksar@ug.edu.ge

**Keywords:** advanced oxidation process, PDS activation, templating synthesis, Rhodamine B, copper loading, water and wastewater treatment

## Abstract

Here, we control the surface activity of hydroxyapatite (HAp) in wastewater treatment which undergoes peroxodisulfate (PDS) activation. Loading the catalytically active Cu species on HAp forms a copper phosphate in the outer layer of HAp. This modification turns a low active HAp into a high catalytically active catalyst in the dye degradation process. The optimal operational conditions were established to be [Cu–THAp]_0_ = 1 g/L, [RhB]_0_ = 20 mg/L, [PDS]_0_ = 7.5 mmol/L, and pH = 3. The experiments indicate that the simultaneous presence of Cu-THAp and PDS synergistically affect the degradation process. Additionally, chemical and structural characterizations proved the stability and effectiveness of Cu-THAp. Therefore, this work introduces a simple approach to water purification through green and sustainable HAp-based materials.

## 1. Introduction

Hydroxyapatite (HAp) is known as one of the most abundant materials on earth, which composes a significant fraction of animals’ bones, fish, shells, and some minerals (e.g., limestone) [1]. HAp is extensively utilized in many industries, including artificial bones [2], biocomposites [3], toothpaste [4], and coatings [5]. In recent decades, HAp has also been an efficient and low-cost material for heterogeneous catalysis, which can even be extracted from biowastes [6,7]. The role of HAp in catalysis is substantial since HAp can contribute to synthesizing cost-effective, stable, and non-toxic catalysts for developing green chemical approaches [8,9]. HAp’s low catalytic activity encourages researchers to modify HAps with catalytically active species. For instance, in one attempt, the surface of HAp was doped with Co species to activate the PDS oxidizing agent in dye removal [10]. PDS activation in water led to the formation of sulfate radicals which have more significant oxidation potential (2.5–3.1 V) and longer half-time (30–40 μs) than other well-known hydroxyl radicals [11].

(Waste)water treatment plays the most critical role in saving the available water reservoir on earth, and the survival of all lives is highly dependent on this magical liquid [12,13]. From the first centuries of human life, the supply of clean and purified water was a challenging issue. In the last two centuries, industrialization and increasing demand for water are the two main reasons for water and its resources’ pollution [14]. Dyes are a class of resistant compounds with significant solubility in aqueous media [15]. Therefore, incomplete degradation is caused if water treatment proceeds with conventional methods such as aeration, coagulation, and filtration [16]. On the other side, advanced oxidation processes (AOP), with their potential to generate reactive oxygen species (ROS), are promising resorts for degrading stable contaminants in water [17]. AOP approaches may include photocatalysis [18,19,20,21,22,23] and sonocatalysis [24,25,26], and electrochemical-based AOPs [27,28] have already been used for water purification. Considering these approaches require energy consumption, free radical utilization methods are the most cost-effective and easy to use on large scales [29]. However, the formation of free radicals in water needs homogenous and heterogeneous catalysis [30,31]. On its own, homogenous catalysis causes the release of some heavy metals, such as Mn and Cr ions, to the solution. Therefore, heterogeneous catalysis is the most environmentally friendly approach compared to the other AOP techniques [32].

Here, an HAp nanostructure is synthesized through a 2D template, i.e., g-C_3_N_4_. Then, the copper ions were loaded on HAp to form copper phosphate on the outer layer and to activate it during catalytic degradation. The synthesized materials were characterized via scanning electron microscopy (SEM), X-ray crystallography (XRD), energy-dispersive X-ray spectroscopy (EDS), and Fourier transform infrared spectroscopy (FTIR). The PDS activation of RhB degradation was selected as the model reaction in this study to optimize variables such as Cu-THAp amount, RhB initial concentration, pH, and PDS initial concentration in the 120 min reaction time. The effects of various synthesized catalysts and radical scavengers were tested through RhB degradation. Additionally, Cu-THAp stability and reusability were investigated in six consecutive RhB degradation cycles. Finally, Cu leaching into the water was analyzed by atomic absorption spectroscopy (AAS), and the reused Cu-THAp sample was characterized again to prove its chemical and morphological stability.

## 2. Materials and Methods

### 2.1. Chemicals

All the chemicals in this research were used as received without any further purification. Melamine (C_3_H_6_N_6_, 99%), cetyltrimethylammonium bromide (CTAB, C_19_H_42_BrN, ≥98%), copper(II) acetate monohydrate (Cu(CO_2_CH_3_)_2_.H_2_O, 99%), calcium nitrate tetrahydrate (Ca(NO_3_)_2_.4H_2_O, 98%), sodium azide (NaN_3_, 99%), diammonium hydrogen phosphate ((NH_4_)_2_HPO_4_, ≥99%), potassium hydroxide (KOH, 99.9%), hydrochloric acid (HCl, 37%), potassium peroxodisulfate (PDS, K_2_S_2_O_8_, ≥99%), RhB (C_28_H_31_ClN_2_O_3_), 2-propanol (IPA, ≥99%), ethanol (C_2_H_5_OH, 99%), *tert*-butanol (t-BuOH, 99.5%), 1,4-benzoquinone (p-BQ, 99%), and methanol (CH_3_OH, ≥99%) were purchased from Merck (Germany).

### 2.2. Characterization

The morphology of materials was studied through the SEM using a Tescan Mira3 microscope (Czech Republic) equipped with EDS and elemental mapping. The crystallographic properties were measured by an X-ray powder diffractometer (Siemens, D5000, Germany). The functional groups on the surface of the synthesized materials were detected by FTIR analysis using a Bruker Tensor 27 spectrophotometer (KBr disk, Germany). Additionally, an atomic absorbance spectrometer (ASS) was used to measure leached Cu amounts in solution using an Analytik Jena novAA^®^ 400 (Germany).

### 2.3. Synthesis

#### 2.3.1. Synthesis of g-C_3_N_4_

A specific amount of melamine was placed in a ceramic boat and transferred into a tubular furnace in the nitrogen atmosphere. Accordingly, melamine was heated up to 550 °C (with heating ramping of 5 °C/min) and kept at that temperature for 4 h to obtain bulk g-C_3_N_4_. For exfoliating bulk g-C_3_N_4_, 0.2 g of it were dispersed in 100 mL DI water and sonicated for 2 h. Afterward, the dispersant was added to a hydrothermal stainless autoclave which was kept at 180 °C for 24 h. The remaining solid was sonicated for 1 h and then centrifuged and washed with DI water and EtOH several times. Finally, the synthesized g-C_3_N_4_ was dried in a vacuum oven.

#### 2.3.2. Synthesis of g-C_3_N_4_-HAp (THAp)

The synthesis of g-C_3_N_4_-templated HAp was followed by the approach reported in the literature [2]. Briefly, the synthesized g-C_3_N_4_ (0.1 g) was sonicated for 2 h in distilled water (100 mL). Afterward, a primary solution including calcium nitrate tetrahydrate (80 mmol) and CTAB was added to the dispersed g-C_3_N_4_ solution and sonicated for an additional 1 h. Separately, diammonium hydrogen phosphate (48 mmol) was dissolved in distilled water (100 mL). Then, the solution was added to the primary solution and stirred while raising the temperature to 90 °C and adjusting the solution’s pH to 11 by KOH. Subsequently, the prepared solution was aged in a hydrothermal process for 2 days at 90 °C. After hydrothermal treatment, the precipitate was obtained after centrifuging at 3000 rpm and washing with distilled water three times. The product was dried at 90 °C for 5 h. The final synthesis stage included drying the product for 6 h to obtain the HAp/g-C_3_N_4_ composite, named THAp.

#### 2.3.3. Synthesis of Cu-Loaded THAp (Cu-THAp)

For loading Cu onto the THAp substrate, copper (II) acetate monohydrate (0.3 mmol) was dissolved in IPA (30 mL) and distilled water mixed solvent (volumetric ratio of 1:1) under sonication for 10 min. Then, THAp (0.5 g) was added to the copper solution and sonicated for 30 min. Subsequently, the suspension was degassed by purging nitrogen for 30 min and stirring for 24 h. Finally, the remaining solid was centrifuged and washed with EtOH and distilled water and then dried in a vacuum drier.

### 2.4. Catalytic RhB Degradation Experiments

A typical RhB catalytic degradation in water was carried out according to the following procedure. First, a RhB solution (20 mg/L, 100 mL) was placed in a 250 mL beaker at room temperature. The pH of the solution was set to 3 using diluted HCl and KOH. Then, Cu-THAp (1 g/L) was added to the RhB contaminated solution and dispersed for 2 min. Subsequently, PDS (7.5 mmol/L) was added to initiate the degradation process in magnetic stirring at 250 rpm. Afterward, 4 mL of the degrading solution was withdrawn at each specific time interval and mixed with 1 mL EtOH to quench the ROS. Then, the solution was filtered through a 0.45 μm syringe filter, and the remaining concentration of RhB in the filtered solution was determined using a Shimadzu^®^ UV–Vis spectrophotometer (Kyoto, Japan) at the λ_max_ of 558 nm. The following formula calculates the degradation efficiency (DE):

DE = [(A_0_ − A_t_)/A_0_] × 100, (1)

where A_0_ is the initial RhB concentration and A_t_ is the RhB concentration after t min of the catalytic degradation process.

## 3. Results and Discussion

### 3.1. Characterization of Synthesized Materials

**Figure 1:**
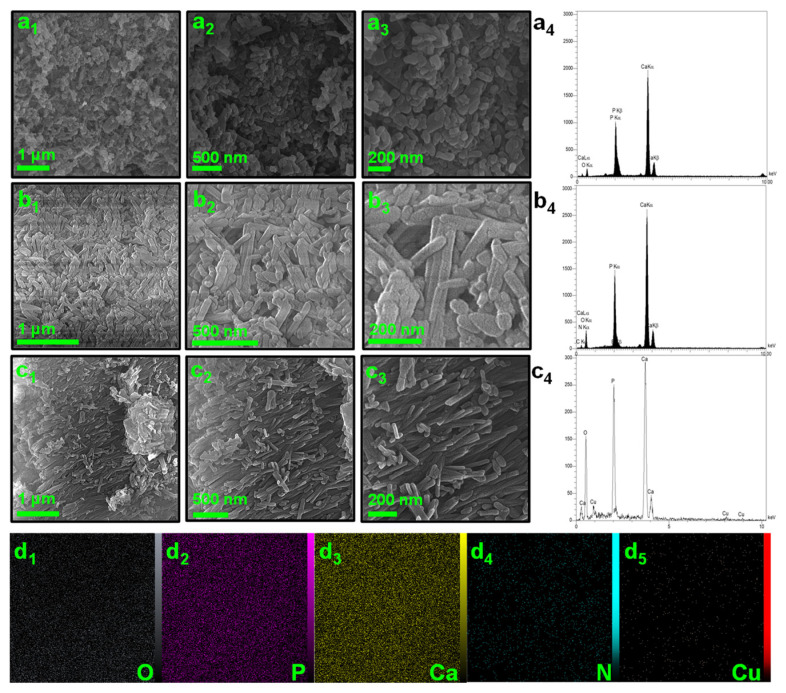
SEM images and corresponding EDS spectra of (**a1**–**a4**) pure HAp, (**b1**–**b4**), THAp, and (**c1**–**c4**) Cu-THAp samples. (**d1**–**d5**) SEM-elemental mapping of Cu-THAp sample.

The following SEM images represent the morphology of the samples. Figure 1a1–a3 shows that the HAp sample is formed from aggregated nanoflakes. However, the addition of g-C_3_N_4_ as a template in HAp synthesis led to the formation of non-ordered THAp nanorods (Figure 1b1–b3). Additionally, when Cu was loaded onto the THAp, the morphology was retained, and Cu did not have a destructive effect on the nanoshape of THAp (Figure 1c1–c3). This process corresponds to the formation of new functional groups during Cu loading on the THAp. The EDS spectrums of HAp, THAp, and Cu-THAp are represented in Figure 1a4–c4, respectively. All expected elements (Ca, P, O, N, and Cu) present sharp peaks in the related energies. Moreover, the Wt.% content of copper in the Cu-THAp samples is about 2.54%. Figure 1d1–d5 show the spatial distribution of each element in the Cu-THAp sample.

**Figure 2:**
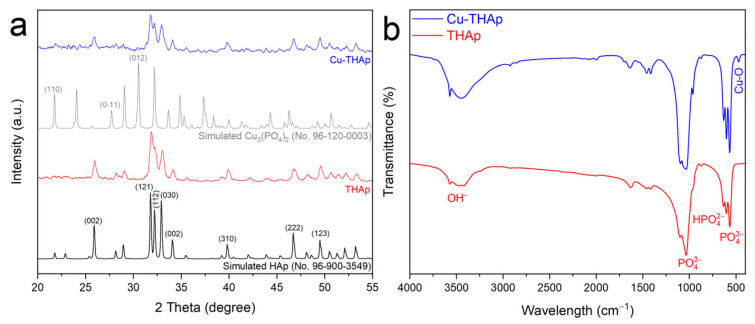
(**a**) XRD patterns and (**b**) FTIR spectrums of samples.

The XRD patterns of the synthesized samples are shown in Figure 2a. The peaks of THAp and Cu-THAp appear in 2θ of 10.82°, 25.90°, 31.80°, 32.15°, 32.89°, 34.18°, 39.88°, 46.76°, and 49.51°, assignable to the (010), (002), (121), (112), (030), (002), (310), (222), and (123) facets of the hexagonal phase in HAp with *P6_3_/m* space group (COD No. 96-900-3549). Adding Cu ions to THAp can lead to an in situ formation of copper phosphate on the surface. By comparing the simulated XRD pattern of copper phosphate (No. 96-120-0003), appearing some major peaks in the 2θ of 21.73°, 27.70°, 30.53°, 37.33°, and 41.32° are attributable to the (110), (011), and (012) planes of the Cu_3_(PO_4_)_2_ with the space group *P-1*. The FTIR spectra of both THAp and Cu-THAp samples are contained from the HAp’s main functional groups (Figure 2b). Hence, the peaks at 3440, 1034, 604, and 565 cm^−1^ can be attributable to OH^−^, ${\mathrm{HPO}}_{4}^{2-}$, and ${\mathrm{PO}}_{4}^{3-}$ functional groups [33]. In the Cu-THAp sample, there is a peak of about 467 cm^−1^. This peak corresponds to the vibrations of the Cu-O bond on the surface of the Cu-THAp sample.

### 3.2. PDS Activation for RhB Catalytic Degradation and Cu-THAp’s Reusability Studies

In this section, the essential operational conditions were studied to determine the optimal point of parameters such as Cu-THAp amount, initial RhB concentrations, pH, and PDS concentrations. All of the mentioned ranges in Table 1 were investigated to achieve the best DE for RhB (as the model contaminant of this work) catalytic degradation.

**Figure 3:**
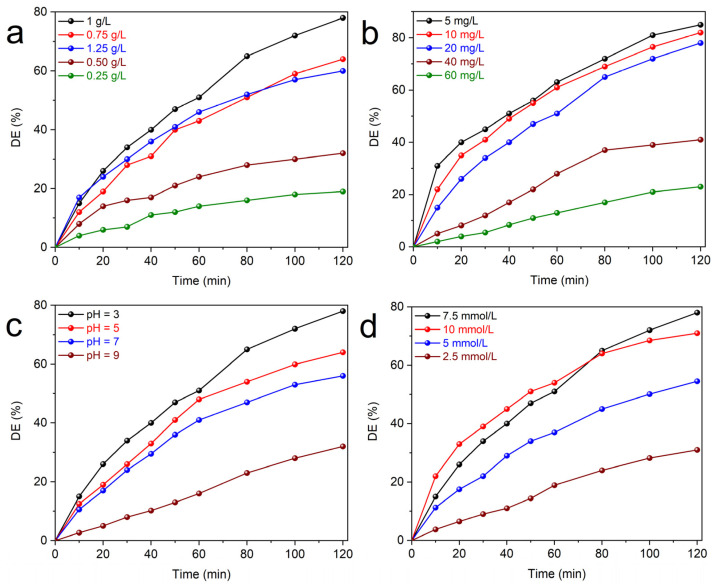
Effect of (**a**) Cu–THAp amount, (**b**) initial RhB concentration, (**c**) pH, and (**d**) PDS concentration on PDS activation for RhB degradation. (Operational conditions: (Cu–THAp)_0_ = 1 g/L, (RhB)_0_ = 20 mg/L, (PDS) = 7.5 mmol/L, and pH = 3).

In the first step, the effect of Cu-THAp amounts was studied in the catalytic degrading 20 mg/L of RhB polluted solution in the presence of 7.5 mmol/L PDS oxidant and pH = 3. As displayed in Figure 3a, a range between 0.25−1.25 g/L of Cu-THAp was studied. The catalyst increasing from 0.25 to 1 g/L led to the enhancement of DE from 18.95 to 78.28%. The more catalyst amount in the solution provides more catalytic active sites for PDS activation. Nevertheless, if its amount crosses over the optimal point, the DE could be diminished because of the aggregation of catalyst particles. Therefore, when the Cu-THAp amount was raised to 1.25 g/L, the DE was decreased to 59.94%. Thus, the catalyst amount of 1 g/L was chosen as the optimum point of this parameter. In the next step, the initial concentration of RhB contaminant was investigated. The ability of a catalyst to degrade a pollutant in low and high concentrations is a critical factor in considering it an efficient and applicable material. In this regard, as depicted in Figure 3b, investigations on a range of RhB initial concentrations between 5 and 60 mg/L were performed to determine the optimum point. High concentrations such as 60 and 40 mg/L had a DE of about 23.07 and 41.10%. The agglomeration of RhB molecules on the catalyst’s surface can be the first reason for this low DE. When the initial concentration was decreased to 20, 10, and 5 mg/L, the subsequent DE was 78.28, 82.15, and 85.46%, respectively. Since the DE presented no salient difference between the mid and low concentrations in the range, the initial RhB concentration of 20 mg/L was selected as the optimum point. The inverse relationship between increasing the initial pollutant concentration and its consequent diminished DE in our batch system is attributed to the constant formation rate of radicals such as ^●^OH and SO_4_^●−^ [11].

pH is a critical factor in the PDS activation system for dye degradation since it not only affects the active radical production rate but also directly affects the surface of the catalyst and the stability of the contaminant. Since PDS is an acidic oxidant, it is more stable in acidic solutions. The SO_4_^●−^ radicals in acidic conditions were formed using Equation (2) [34]:

(2) SO42-+O•H+H+ →  SO4•-+H2O.

As shown in Figure 3c, an acidic pH of 3 was more efficient for RhB degradation, and by increasing the pH to 5, 7, and 9, the DE was reduced to 64.05, 55.80, and 31.76%, respectively. The generation of di-anion forms of PDS at higher pH values was the main factor in the reduction of its degradation efficiency [35]. Therefore, the acidic condition of pH = 3 was utilized in all experiments. As the last parameter of the operational condition, the PDS concentration effect was investigated during RhB degradation. A set of concentrations from 2.5 to 10 mmol/L was selected to test under the condition of (Cu–THAp)_0_ = 1 g/L, (RhB)_0_ = 20 mg/L, and pH = 3 (Figure 3d). In this regard, when the PDS concentration was set to 2.5 mmol/L, a DE of 31.00% was observed. Subsequently, PDS concentrations of 5 and 7.5 mmol/L showed DE = 54.51 and 78.28%, respectively. However, the PDS concentration of 10 mmol/L had a diminished DE of 71.25%, which could be justified by the elimination of produced sulfate radicals via PDS molecules [36].

**Figure 4:**
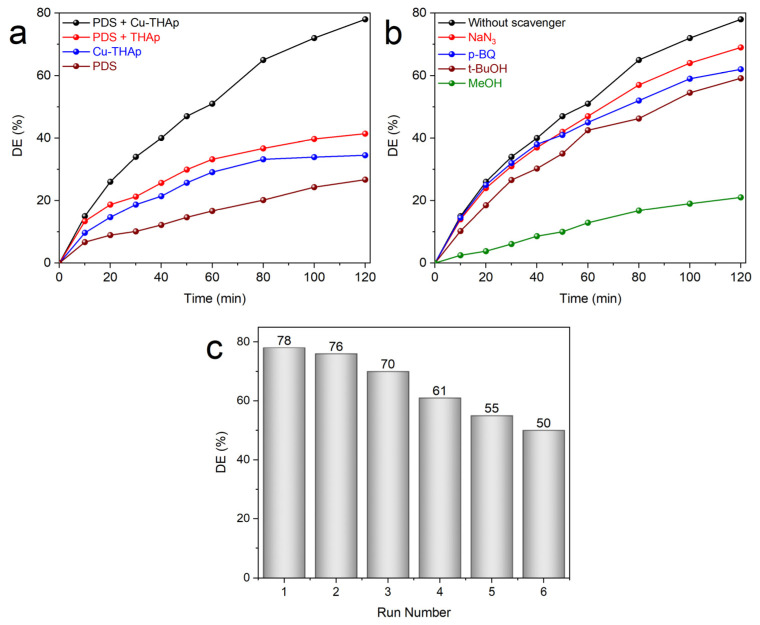
Effect of (**a**) various processes and (**b**) radicals’ scavengers on PDS activation for RhB degradation. (**c**) Reusability of CU-THAp during PDS activation for RhB degradation in six consecutive cycles. (Operational conditions: (Cu–THAp)_0_ = 1 g/L, (RhB)_0_ = 20 mg/L, PDS = 7.5 mmol/L, and pH = 3).

Various processes were tested to evaluate their individual and combined performance in RhB degradation through optimal operational conditions. As displayed in Figure 4a, the utilization of bare PDS in solution was just able to degrade 26.75% of RhB. Furthermore, usage of Cu-THAp (adsorption process) led to 34.53% of DE. In combined processes, two forms of (THAp + PDS) and (Cu-THAp + PDS) had a DE of 41.56 and 78.28%, respectively. The existence of a 36.72% difference between these processes was proof of more generation of active radicals on the surface of THAp when it was loaded with copper particles. The synergy factor is a numerical value used to show the success of the process’s combination. When the synergy factor is above 1, the combination is successful. This value is calculated by applying the pseudo-first-order kinetic model to each process and determining their apparent kinetic rate constants (k_app_, with R^2^ ≥ 99.99). In this way, the k_app_ was calculated to be about 0.0124, 0.0034, and 0.0024 1/min for combined Cu-THAp + PDS, bare Cu-THAp, and bare PDS processes, respectively. The synergy factor can be determined by Equation (3) [37]:

(3) $$\mathrm{Synergy}\;\mathrm{factor}=\frac{k_{\mathrm{app}}\;(\mathrm{Cu}-\mathrm{THAp}/\mathrm{PDS})}{k_{\mathrm{app}}{\;(\mathrm{Cu}-\mathrm{THAp})+k}_{\mathrm{app}}\;\left(\mathrm{PDS}\right)\;}.$$

Hence, the synergy factor is calculated to be 2.14. Recently, the degradation turnover (dTON) as a numerical value has been introduced as an easy tool to judge catalytic degradation systems [37]. The dTON of the control experiment was estimated to be 2.05 µmol h^−1^ g_cat_^−1^.

Detecting the ROS is essential in AOP applications. The radical scavenger test is an effective tool to identify each radical cooperation in the degradation process. Here, the RhB:scavenger ratio was estimated to be 1:75 as the optimal conditions. As shown in Figure 4b, NaN_3_, p-BQ, t-BuOH, and MeOH were utilized as scavengers in each one of ^1^O_2_^●^, O_2_^●−^, ^●^OH, and both (^●^OH and SO_4_^●−^), respectively [38]. The utilization of these scavengers decreased the DE from 78.28% to 69.34, 62.47, 59.14, and 21.68% in the presence of NaN_3_, p-BQ, t-BuOH, and MeOH, respectively. Therefore, SO_4_^●−^ radicals are composing the main active radicals during RhB degradation. The reusability of each catalyst is a promising sign of its scale-up and industrial-based application. In this regard, Cu-THAp was applied in six consecutive RhB degradation cycles. Figure 4c shows that the DE diminished by about 28% after six cycles. Since the Cu(II)/Cu(III) redox pair is key to the activation of PDS on the Cu-THAp surface, this diminished DE is justifiable. The leaching of Cu into the solution and agglomeration of RhB molecules on the Cu-THAp surface can be introduced as the most important phenomenon for this decreased efficiency. Since environmental issues have a great impact on the earth’s ecosystem, the leached amount of copper in the solution was determined and recorded in Table 2. The World Health Organization (WHO) set the threshold of Cu in drinking water at 1.00 mg/L (WHO, *Guidelines for drinking-water quality*, 4th edition, 2011). Therefore, the leached Cu amounts added to the solution during the six RhB degradation cycles are under the requested limit by WHO.

**Figure 5:**
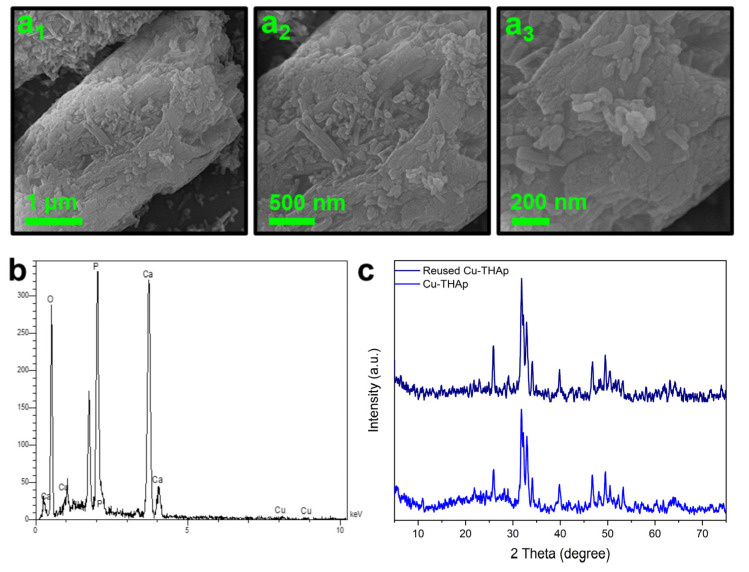
(**a1**–**a3**) SEM images, (**b**) EDS spectra, and (**c**) XRD pattern of reused Cu-THAp.

The stability of Cu-THAp was studied via SEM, EDS, and XRD analysis after six consecutive RhB degradation cycles. The SEM images shown in Figure 5a1–a3 indicate the adsorption of THAp nanorods after six RhB degradation cycles. However, the crystalline structure of THAp was retained, and only its hieratical and uniform order was damaged. Based on the EDSF data, the Cu Wt.% content was decreased to 1.65 (Figure 5b). The XRD pattern of reused Cu-THAp indicates its structural preservability after six consecutive cycles (Figure 5c). Compared to other catalysts used in AOPs, our proposed catalyst is free of noble metal, easy to synthesize, low-cost, and has no rare earth metal [39,40,41].

Finally, PDS’s activation mechanism using hydroxyapatite/copper phosphate is discussed. Several reports support that the formation of Cu(III) intermediate in the presence of PDS is a key for proceeding with degradation; however, we hypothesize the degradation process through the formation of various oxidation states of copper species which achieve the Cu(II)/Cu(III) cyclic redox process and hence, catalytic degradation [42,43]. Since E^o^_Cu(III)/Cu(II)_ is equal to 2.4, Cu(III) might be rapidly reduced by RhB or its generated intermediates with lower redox potentials in the degradation system [44,45]. However, we have no in situ results to confirm this claim. Briefly, Equations (4)–(6) can express the activation and degradation mechanism.

(4) $${\mathrm{Cu}(\mathrm{II})+S}_{2}O_{8}^{2-}\;\overset{\;}{\to }{\;\mathrm{Cu}\;(\mathrm{III})+\mathrm{SO}}_{4}^{•-}{+\mathrm{SO}}_{4}^{2-}.$$

(5) $$\mathrm{Cu}\left(\mathrm{III}\right){+S}_{2}O_{8}^{2-}{+2H}_{2}O\;\overset{\;}{\to }{\;\mathrm{Cu}\;(I)+2\mathrm{HSO}}_{4}^{-}{+O}_{2}^{•-}{+2H}^{+}.$$

(6) SO4•-+O•H+O2•-+RhB→  Degraded products.

## 4. Conclusions

In conclusion, HAp nanostructures were morphologically and crystally tuned using g-C_3_N_4_. Copper’s coordination with the hydroxyapatite surface was proved by FTIR spectroscopy, and this copper species acted as an active catalyst site in dye degradation. The synthesized materials were characterized before and after catalytic application. In the experiment, RhB dye degradation was carried out thoroughly through the catalytic role of Cu-THAp, which was performed by PDS activation. Since the structure of copper phosphate is highly stable, it is predicted that the loaded Cu species would form a viable copper phosphate structure on the surface of hydroxyapatite and act as the active catalytic centers. The synergy factor for the proposed degradation system is estimated to be 2.14. The scavengers’ studies indicate more activity of THAp-induced SO_4_^●−^ radicals in degradation reactions. This study offers a novel, green, reusable, stable, and economical HAp-based catalyst water and wastewater treatment application through AOP techniques.

## Figures and Tables

**Table 1:** Parameters and their related ranges to find optimal operational conditions.

Parameter	Range
Cu-THAp amount (g/L)	0.25–1.25
RhB Initail concentration (mg/L)	5–60
pH	3–9
PDS concentration (mmol/L)	2.5–10

**Table 2:** Cu leaching to the aqueous solution.

Run Number	Cu Concentration (mg/L)
Blank sample	0.01
1	0.58
2	0.53
3	0.47
4	0.49
5	0.41
6	0.34

## Data Availability

Data are available in the main text.

## References

[1] Mohd Pu’ad N.A.S., Koshy P., Abdullah H.Z., Idris M.I., Lee T.C. (2019). Syntheses of hydroxyapatite from natural sources. Heliyon.

[2] Doustkhah E., Najafi Zare R., Yamauchi Y., Taheri-Kafrani A., Mohtasham H., Esmat M., Ide Y., Fukata N., Rostamnia S., Sadeghi M.H. (2019). Template-oriented synthesis of hydroxyapatite nanoplates for 3D bone printing. J. Mater. Chem. B.

[3] Zhang J., Huang D., Liu S., Dong X., Li Y., Zhang H., Yang Z., Su Q., Huang W., Zheng W. (2019). Zirconia toughened hydroxyapatite biocomposite formed by a DLP 3D printing process for potential bone tissue engineering. Mater. Sci. Eng. C.

[4] O’Hagan-Wong K., Enax J., Meyer F., Ganss B. (2022). The use of hydroxyapatite toothpaste to prevent dental caries. Odontology.

[5] Habibovic P., Barrère F., Van Blitterswijk C.A., de Groot K., Layrolle P. (2002). Biomimetic Hydroxyapatite Coating on Metal Implants. J. Am. Ceram. Soc..

[6] Maschmeyer T., Luque R., Selva M. (2020). Upgrading of marine (fish and crustaceans) biowaste for high added-value molecules and bio(nano)-materials. Chem. Soc. Rev..

[7] Meng J., Pan W., Gu T., Bu C., Zhang J., Wang X., Liu C., Xie H., Piao G. (2021). One-Pot Synthesis of a Highly Active and Stable Ni-Embedded Hydroxyapatite Catalyst for Syngas Production via Dry Reforming of Methane. Energy Fuels.

[8] El-Hosainy H., Mine S., Toyao T., Shimizu K.-i., Tsunoji N., Esmat M., Doustkhah E., El-Kemary M., Ide Y. (2022). Layered silicate stabilises diiron to mimic UV-shielding TiO_2_ nanoparticle. Mater. Today Nano.

[9] Esmat M., Doustkhah E., Abdelbar M., Tahawy R., El-Hosainy H., Abdelhameed M., Ide Y., Fukata N. (2022). Structural Conversion of Cu-Titanate into Photoactive Plasmonic Cu-TiO_2_ for H_2_ Generation in Visible Light. ACS Sustain. Chem. Eng..

[10] Pang Y., Kong L., Chen D., Yuvaraja G., Mehmood S. (2020). Facilely synthesized cobalt doped hydroxyapatite as hydroxyl promoted peroxymonosulfate activator for degradation of Rhodamine B. J. Hazard. Mater..

[11] Jamal Sisi A., Fathinia M., Khataee A., Orooji Y. (2020). Systematic activation of potassium peroxydisulfate with ZIF-8 via sono-assisted catalytic process: Mechanism and ecotoxicological analysis. J. Mol. Liq..

[12] He C., Liu Z., Wu J., Pan X., Fang Z., Li J., Bryan B.A. (2021). Future global urban water scarcity and potential solutions. Nat. Commun..

[13] Ricart S., Villar-Navascués R.A., Hernández-Hernández M., Rico-Amorós A.M., Olcina-Cantos J., Moltó-Mantero E. (2021). Extending Natural Limits to Address Water Scarcity? The Role of Non-Conventional Water Fluxes in Climate Change Adaptation Capacity: A Review. Sustainability.

[14] Dolan F., Lamontagne J., Link R., Hejazi M., Reed P., Edmonds J. (2021). Evaluating the economic impact of water scarcity in a changing world. Nat. Commun..

[15] Zhou C., Xia W., Huang D., Cheng M., Zhang H., Cai T., Xiong W., Yang Y., Song B., Wang W. (2021). Strategies for enhancing the perylene diimide photocatalytic degradation activity: Method, effect factor, and mechanism. Environ. Sci. Nano.

[16] Zamora-Ledezma C., Negrete-Bolagay D., Figueroa F., Zamora-Ledezma E., Ni M., Alexis F., Guerrero V.H. (2021). Heavy metal water pollution: A fresh look about hazards, novel and conventional remediation methods. Environ. Technol. Innov..

[17] Isgoren M., Gengec E., Veli S., Hassandoost R., Khataee A. (2021). The used automobile catalytic converter as an efficient catalyst for removal of malathion through wet air oxidation process. Int. J. Hydrog. Energy.

[18] Hassandoost R., Pouran S.R., Khataee A., Orooji Y., Joo S.W. (2019). Hierarchically structured ternary heterojunctions based on Ce^3+^/ Ce^4+^ modified Fe_3_O_4_ nanoparticles anchored onto graphene oxide sheets as magnetic visible-light-active photocatalysts for decontamination of oxytetracycline. J. Hazard. Mater..

[19] Doustkhah E., Esmat M., Fukata N., Ide Y., Hanaor D.A.H., Assadi M.H.N. (2022). MOF-derived nanocrystalline ZnO with controlled orientation and photocatalytic activity. Chemosphere.

[20] Cheng T., Gao H., Liu G., Pu Z., Wang S., Yi Z., Wu X., Yang H. (2022). Preparation of core-shell heterojunction photocatalysts by coating CdS nanoparticles onto Bi_4_Ti_3_O_12_ hierarchical microspheres and their photocatalytic removal of organic pollutants and Cr(VI) ions. Colloids Surf. Physicochem. Eng. Asp..

[21] Xiao L., Youji L., Feitai C., Peng X., Ming L. (2017). Facile synthesis of mesoporous titanium dioxide doped by Ag-coated graphene with enhanced visible-light photocatalytic performance for methylene blue degradation. RSC Adv..

[22] Li L., Gao H., Liu G., Wang S., Yi Z., Wu X., Yang H. (2022). Synthesis of carnation flower-like Bi_2_O_2_CO_3_ photocatalyst and its promising application for photoreduction of Cr(VI). Adv. Powder Technol..

[23] Chen P., Liu F., Ding H., Chen S., Chen L., Li Y.-J., Au C.-T., Yin S.-F. (2019). Porous double-shell CdS@C_3_N_4_ octahedron derived by in situ supramolecular self-assembly for enhanced photocatalytic activity. Appl. Catal. B Environ..

[24] Ansarian Z., Khataee A., Arefi-Oskoui S., Orooji Y., Lin H. (2022). Ultrasound-assisted catalytic activation of peroxydisulfate on Ti_3_GeC_2_ MAX phase for efficient removal of hazardous pollutants. Mater. Today Chem..

[25] Yousef Tizhoosh N., Khataee A., Hassandoost R., Darvishi Cheshmeh Soltani R., Doustkhah E. (2020). Ultrasound-engineered synthesis of WS_2_@CeO_2_ heterostructure for sonocatalytic degradation of tylosin. Ultrason. Sonochem..

[26] Khataee A., Hassandoost R., Rahim Pouran S. (2018). Cerium-substituted magnetite: Fabrication, characterization and sonocatalytic activity assessment. Ultrason. Sonochem..

[27] Ahmadi A., Zarei M., Hassani A., Ebratkhahan M., Olad A. (2021). Facile synthesis of iron(II) doped carbonaceous aerogel as a three-dimensional cathode and its excellent performance in electro-Fenton degradation of ceftazidime from water solution. Sep. Purif. Technol..

[28] Ebratkhahan M., Zarei M., Babaei T., Hosseini M.G., Hosseini M.M., Fathipour Z. (2022). Efficient electrochemical removal of 5-fluorouracil pharmaceutical from wastewater by mixed metal oxides via anodic oxidation process. Chemosphere.

[29] Wang W., Chen M., Wang D., Yan M., Liu Z. (2021). Different activation methods in sulfate radical-based oxidation for organic pollutants degradation: Catalytic mechanism and toxicity assessment of degradation intermediates. Sci. Total Environ..

[30] Yu F., Poole III D., Mathew S., Yan N., Hessels J., Orth N., Ivanović-Burmazović I., Reek J.N.H. (2018). Control over Electrochemical Water Oxidation Catalysis by Preorganization of Molecular Ruthenium Catalysts in Self-Assembled Nanospheres. Angew. Chem. Int. Ed..

[31] Cao W., Lin L., Qi H., He Q., Wu Z., Wang A., Luo W., Zhang T. (2019). In-situ synthesis of single-atom Ir by utilizing metal-organic frameworks: An acid-resistant catalyst for hydrogenation of levulinic acid to γ-valerolactone. J. Catal..

[32] Zheng M., Ding Y., Cao X., Tian T., Lin J. (2018). Homogeneous and heterogeneous photocatalytic water oxidation by polyoxometalates containing the most earth-abundant transition metal, iron. Appl. Catal. B Environ..

[33] Gheisari H., Karamian E., Abdellahi M. (2015). A novel hydroxyapatite –Hardystonite nanocomposite ceramic. Ceram. Int..

[34] Wang S., Zhou N. (2016). Removal of carbamazepine from aqueous solution using sono-activated persulfate process. Ultrason. Sonochem..

[35] Kolthoff I.M., Miller I.K. (1951). The Chemistry of Persulfate. I. The Kinetics and Mechanism of the Decomposition of the Persulfate Ion in Aqueous Medium1. J. Am. Chem. Soc..

[36] Wang C.-W., Liang C. (2014). Oxidative degradation of TMAH solution with UV persulfate activation. Chem. Eng. J..

[37] Hassandoost R., Kotb A., Movafagh Z., Esmat M., Guegan R., Endo S., Jevasuwan W., Fukata N., Sugahara Y., Khataee A. (2022). Nanoarchitecturing bimetallic manganese cobaltite spinels for sonocatalytic degradation of oxytetracycline. Chem. Eng. J..

[38] Sisi A.J., Khataee A., Fathinia M., Vahid B. (2020). Ultrasonic-assisted degradation of a triarylmethane dye using combined peroxydisulfate and MOF-2 catalyst: Synergistic effect and role of oxidative species. J. Mol. Liq..

[39] Hasija V., Raizada P., Sudhaik A., Sharma K., Kumar A., Singh P., Jonnalagadda S.B., Thakur V.K. (2019). Recent advances in noble metal free doped graphitic carbon nitride based nanohybrids for photocatalysis of organic contaminants in water: A review. Appl. Mater. Today.

[40] Keerthana S.P., Yuvakkumar R., Kumar P.S., Ravi G., Velauthapillai D. (2021). Rare earth metal (Sm) doped zinc ferrite (ZnFe2O4) for improved photocatalytic elimination of toxic dye from aquatic system. Environ. Res..

[41] Mohammadi S., Esmailpour A., Doustkhah E., Assadi M.H.N. (2022). Stability Trends in Mono-Metallic 3d Layered Double Hydroxides. Nanomaterials.

[42] Chen J., Zhou X., Zhu Y., Zhang Y., Huang C.-H. (2020). Synergistic activation of peroxydisulfate with magnetite and copper ion at neutral condition. Water Res..

[43] Arfanis M.K., Athanasekou C.P., Sakellis E., Boukos N., Ioannidis N., Likodimos V., Sygellou L., Bouroushian M., Kontos A.G., Falaras P. (2019). Photocatalytic properties of copper—Modified core-shell titania nanocomposites. J. Photochem. Photobiol. A Chem..

[44] Li W., Liu B., Wang Z., Wang K., Lan Y., Zhou L. (2020). Efficient activation of peroxydisulfate (PDS) by rice straw biochar modified by copper oxide (RSBC-CuO) for the degradation of phenacetin (PNT). Chem. Eng. J..

[45] Feng S., Xiao B., Wu M., Wang Y., Chen R., Liu H. (2020). Copper phosphide: A dual-catalysis-center catalyst for the efficient activation of peroxydisulfate and degradation of Orange II. Sep. Purif. Technol..

